# Supplementary material for: miR403a and SA Are Involved in NbAGO2 Mediated Antiviral Defenses Against TMV Infection in Nicotiana benthamiana
Source: Genes (Basel). 2019 Jul 12;10(7):526. doi: 10.3390/genes10070526 (PMC6679004; doi:10.3390/genes10070526)
Supplement: Supplementary file 1 [file genes-10-00526-s001.pdf]

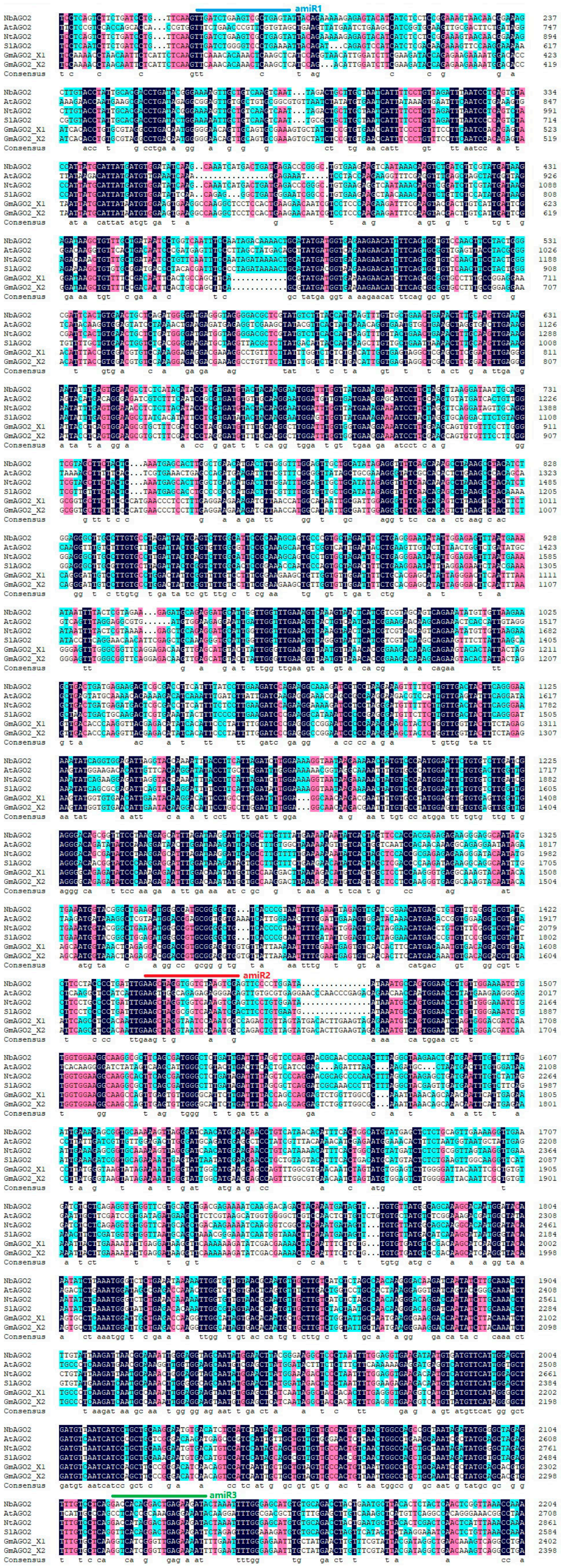

Figure S1: ***NbAGO2*** **(Unigene No.** Nbv3K585706870, retrieved from www.benthgenome.com) was aligned with *Arabidopsis thaliana NbAGO2* (Accession No. NM_102866.3), *Nicotiana tabacum NtAGO2* (Accession No. XM_016619080.1), *Solanum lycopersicum SlyAGO2* (Accession No. NM_001279306.2), and *Glycine max GmAGO2* (Accession No. XM_006605460.3) by DNAMAN software (Version

6.0.3.99, Lynnon Biosoft, San Ramon, CA, USA). The sequences for amiR target sites were shown on top by lines with different colors.

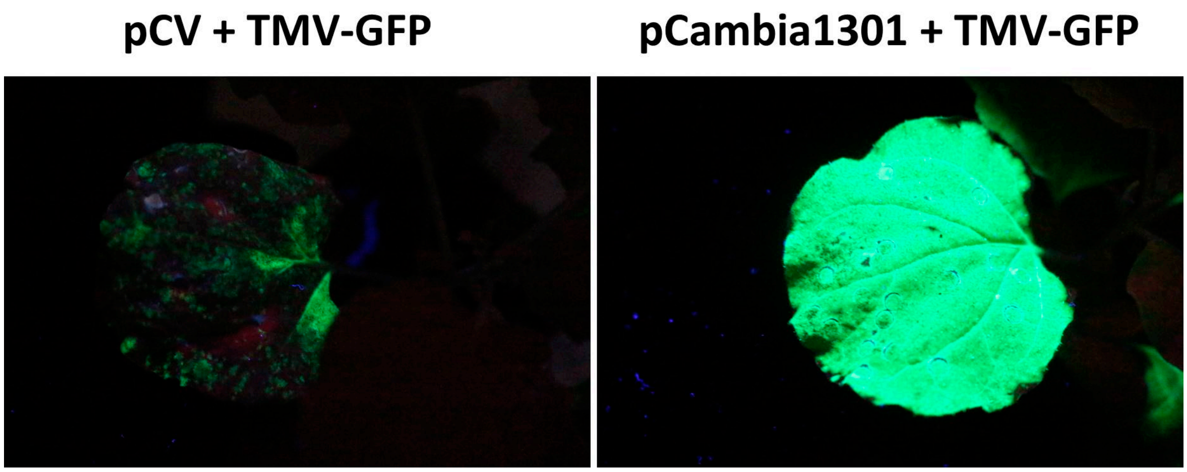

Figure 2. *N. benthamiana* leaves were first inoculated with pCV (both pCVA and pCVB), and then were infected with TMV-GFP (pJL24) one day later. As a control, pCambia1301, the backbone of pCV infectious clone used here, was inoculated. The result showed that first inoculation of pCV impeded TMV-GFP infections.

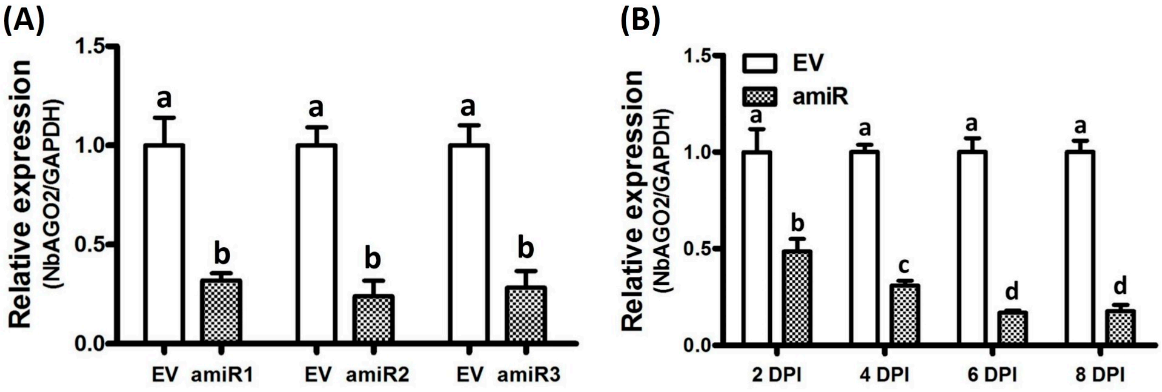

Figure S3: The experiments in (**A**) and (**B**) were performed the same as Figure 1 and 2, respectively**.** The only difference is *Actin* gene was used as internal control here instead of *GAPDH* in Figure 1 and 2.

```
NbAgo2   5'  U G U U U G U G C G U G A A U C U A A  3'
                 | | | | | | | | | | | | | | | | | |
miR403a  3'  U C A A A C A C G C A C U U A G A U U  5'
```

Figure S4: Sequence alignment between miR403a and target *NbAGO2* gene from 17Wt *Nicotiana Benthamiana*. "|" indicates perfect match, while gap shows mismatches.
